# eConsent administered by Community Health Workers in a study using the Trials within Cohorts (TwiCs) design—Experiences from the Community-Based chronic Care Lesotho (ComBaCaL) project

**DOI:** 10.1177/20552076241288757

**Published:** 2024-10-01

**Authors:** Felix Gerber, Thesar Tahirsylaj, Thabo Ishmael Lejone, Tristan Lee, Giuliana Sanchez-Samaniego, Fabian Raeber, Sesale Masike, Ravi Gupta, Manthabiseng Molulela, Makhebe Khomolishoele, Mota Mota, Matumaole Bane, Mamoronts’ane Pauline Sematle, Retselisitsoe Makabateng, Jason Immanuel Browne, Jonas Wittwer, Dave Brian Basler, Kevin Kindler, Niklaus Daniel Labhardt, Alain Amstutz

**Affiliations:** 1 Division of Clinical Epidemiology, Department of Clinical Research, 30262 University Hospital Basel, Basel, Switzerland; 2 27209 Faculty of Medicine, University of Basel, Basel, Switzerland; 3 30247 Department of Medicine, Swiss Tropical and Public Health Institute, Allschwil, Switzerland; 4 561113 SolidarMed, Maseru, Lesotho; 5 Faculty of Business, Economics and Informatics, 27217 University of Zurich, Zurich, Switzerland; 6 Oslo Center for Biostatistics and Epidemiology, Oslo University Hospital, 6305 University of Oslo, Oslo, Norway; 7 152331 Bristol Medical School, University of Bristol, Bristol, UK

**Keywords:** Electronic consent, Community Health Worker, clinical decision support system, Trials within Cohorts, Lesotho

## Abstract

Improving access to essential health services requires the development of innovative health service delivery models and their scientific assessment in often large-scale pragmatic trials. In many low- and middle-income countries, lay Community Health Workers (CHWs) play an important role in delivering essential health services. As trusted members of their communities with basic medical training, they may also contribute to health data collection. Digital clinical decision support applications may facilitate the involvement of CHWs in service delivery and data collection. Electronic consent (eConsent) can streamline the consent process that is required if the collected data is used for the scientific purposes. Here, we describe the experiences of using eConsent in the Community-Based chronic Care Lesotho (ComBaCaL) cohort study and multiple nested pragmatic cluster-randomized trials assessing CHW-led care delivery models for type 2 diabetes and arterial hypertension using the Trials within Cohorts (TwiCs) design. More than a hundred CHWs, acting both as service providers and data collectors in remote villages of Lesotho utilize an eConsent application that is linked to a tailored clinical decision support and data collection application. The eConsent application presents simplified consent information and generates personalized consent forms that are signed electronically on a tablet and then uploaded to the database of the clinical decision support application. This significantly streamlines the consent process and allows for quality consent documentation through timely central monitoring, facilitating the CHW-led management of a large-scale population-based cohort in a remote low-resource area with continuous enrollment—currently at more than 16,000 participants.

For important communicable and noncommunicable diseases such as HIV/AIDS, arterial hypertension and diabetes, tools for effective diagnosis, prevention and treatment are available. However, particularly in low resource settings, due to insufficient access to these essential health services, rates of avoidable early morbidity and mortality related to these conditions remain unacceptably high. The evidence required for service delivery models to bring those tools to people most in need is often generated in large-scale pragmatic trials.^1^

## Community Health Workers in service delivery and research

In Lesotho and many other countries in Southern Africa, Community Health Workers (CHWs, called Village Health Workers in Lesotho), lay community members who are elected and trained to provide basic healthcare services in their villages, are an established cadre in the healthcare system linking their communities to often hard-to-reach health facilities.^2^ Decentralization of healthcare services through task-shifting to CHWs may be key to improving access to essential health services. Smartphone- or tablet-based digital clinical decision support systems may facilitate the task-shifting to CHWs through clinical guidance and remote monitoring.^3,4^ In addition to service delivery, CHWs may play a pivotal role in population-based data collection, especially in remote rural areas, since they are a trusted cadre permanently residing in the community.^5^ Involving CHWs at scale in research data collection is challenging from a logistical, ethical and data quality perspective. Main challenges are linked to the limited research expertise of CHWs which may lead to shortcomings in protocol adherence, completeness and accuracy of data and inadequate consent and enrolment procedures.^6,7^ Furthermore, the supply management of medical equipment or paper-based forms as well as monitoring of study procedures are complex, especially if CHWs operate in remote villages. Digitalization, including the use of electronic data capture systems in combination with electronic consent (eConsent) may mitigate some of these challenges and be a key enabler for efficient population-level research in remote settings.

## Trials within Cohorts

Trials within Cohorts (TwiCs) is a randomized trial design that may considerably increase the efficiency of pragmatic trials in cohort studies. Participants are recruited into a prospective cohort and give their consent not only to regular data collection at cohort visits, but also to be randomly sampled for future low-risk pragmatic trials.^8,9^ Hence, the cohort infrastructure and data are used for multiple randomized controlled trials that would otherwise require the set-up of separate infrastructure. The consent information for newly enrolled participants into a cohort with nested TwiCs must contain information on cohort data collection and future TwiCs randomizations at the same time. It is thus usually more complex than consent information for traditional randomized trials, requiring careful communication of consent information.^10^

Here, we report the experiences of developing and using eConsent in combination with a clinical decision support application by CHWs in the Community-Based chronic Care Lesotho (ComBaCaL) cohort (NCT05596773) using the TwiCs design.

## Objectives, population and design

The open, prospective ComBaCaL cohort is located in 103 randomly selected villages in the rural Northeast of Lesotho, a small, mountainous country surrounded by South Africa. The objective of the ComBaCaL cohort is to generate evidence on the burden, and risk factors of chronic diseases, including arterial hypertension, diabetes and HIV in the rural areas of Lesotho. Furthermore, the cohort is designed to serve as a platform for the implementation of randomized, pragmatic TwiCs assessing the effectiveness of community-based, CHW-led chronic disease care interventions. ComBaCaL is a population-based cohort and all people living in one of the study villages are eligible for participation. Information on chronic disease risk factors, HIV, diabetes and arterial hypertension prevalence, and treatment status will be reported in a separate manuscript. The cohort enrolment started in February 2023. By June 2024, 16,461 individuals from 5,274 households were asked for consent to participate in the study. For 5,263 households (99.8%) with 16,430 individuals, household consent was obtained, while 11 households (0.2%) with 31 people refused household consent. Out of the 16,430 people eligible for individual consent, 67 people (0.4%) refused individual consent and for 205 people (1.2%), the consent was pending at the time of submission of this manuscript. In total, 16,158 participants (98.3%) gave individual consent. In June 2024, 14,737 (91.2%) of the 16,158 consenting participants were retained in the cohort, while 1,119 (6.9%) have moved out of their village, 189 (1.2%) withdrew consent, and 113 (0.7%) died since enrolment. Three nested TwiCs are ongoing, assessing the effectiveness of community-based diabetes^11^ (NCT05743387) and arterial hypertension care^12^ (NCT05684055) delivered by CHWs. The TwiCs will be completed in 2025 with results being reported in separate manuscripts.

## Clinical decision support and eConsent

In each ComBaCaL cohort village, there is one trained CHW equipped with a tablet on which a tailored clinical decision support and data collection application (ComBaCaL app) and a specifically developed consent application (eConsent app) are installed. The ComBaCaL app is based on the open source Community Health Toolkit (CHT) framework^13^ and guides the CHWs through all processes including registration of participants, regular cohort data collection, trial assessments and health service delivery based on clinical algorithms. CHWs visit participants at their home. Consent is asked at three different levels. First, oral consent at cluster level was obtained from the village chief before start of the study in each village. Second, oral consent at household level is obtained from the household head or a representative before household members are registered in the ComBaCaL app. Third, written consent at individual level is asked using the eConsent application described here. After the initial registration of a person in the ComBaCaL app, an electronic report form that contains a link to the eConsent app is triggered. In the eConsent app, CHWs input the participant's name, age, preferred language (Sesotho or English) and whether the participant is literate. Based on this information, the appropriate version of the study information document is automatically displayed in a simplified text version that is read out or shown to the participant. Thereafter, a file in portable document format (PDF) with the full study information is generated containing the current date and name of the participant (and the names of the guardian and/or witness if applicable). This personalized informed consent PDF is then signed electronically with the finger on the tablet screen by the participant, the witness and/or guardian if applicable, and the CHW. Illiterate participants give consent with a cross, electronically countersigned by a witness on the same document. For children (younger than 10 years) and people incapable of judgment, a guardian consent is required and for adolescents (10 to 17 years) a guardian consent together with the adolescent assent. After signature, the consent PDF is saved on the tablet. The CHW is automatically referred back to the report form in the ComBaCaL app where the signed PDF can be linked to the participant's electronic record. No formal verification of participants’ identity documents is done as the consent is taken during a personal encounter with the CHW, who knows the participants personally.

The consent form was designed in collaboration with the local study team consisting of nurses living in the study area who have large experience in working with the local communities and in supervising and mentoring CHWs. The consent form was revised after inputs from the CHWs during the training and reviewed and approved by the study steering committee that included a community representative, local ministry of health officials and researchers from the local nongovernmental implementing organization SolidarMed (www.solidarmed.ch). The consent is part of the decision support algorithm of the ComBaCaL app, and only after upload of the signed PDF health data collection is permitted. This process runs without access to internet, a crucial aspect in our setting with recruitment and data collection in rural homes. Once internet is available, the ComBaCaL app automatically synchronizes and the consent PDF is uploaded to the study database, hosted on a secure server at the University Hospital Basel, Switzerland, together with all other report forms. If a participant withdraws consent, the CHW documents this in the ComBaCaL app, which leads to the automatic deactivation of the participant's profile in the app preventing further data collection.

## Monitoring and archiving

For quality checks, the consent PDFs are downloaded from the server on a weekly basis. Using automated data quality checks programmed in the statistical software R,^14^ all consent forms are screened for potential errors (missing signatures, name spelling errors, wrong consent version). Queries are followed up by the study team. This approach allows for timely identification of errors and re-training of CHWs and re-consenting of participants if indicated. After confirmation that the version and all signatures are correct, the consent PDF is moved from the database of the ComBaCaL app to a separate archive on a secure server at the University Hospital Basel to avoid slowing down of the ComBaCaL app due to the large data size of the PDF documents.

## Discussion

Large-scale, pragmatic trials are keys for the evidence-based improvement of health service delivery models. However, conducting such trials is costly and logistically challenging, especially in remote areas, and solutions to streamline trial implementation are needed.

In the ComBaCaL study, the TwiCs design, the use of interlinked clinical decision support and eConsent applications, as well as the dual function of CHWs as service providers and data collectors, contribute significantly to efficient high quality study implementation. Using an electronic signature eliminates the need for printing large amounts of consent forms, including all possible version scenarios, and then distributing and collecting the forms to and from more than 100 remote villages. Most importantly, the upload of electronically signed consent PDFs to the study database significantly enhances the quality of the consent documentation as it prevents loss of documents and enables timely and efficient quality control. Furthermore, embedding the consent in the clinical decision support application ensures that no data is collected without prior consent. However, uploading large amounts of PDF files to the clinical decision support application requires regular archiving of PDFs after quality checks to avoid slowing down of the clinical decision support application due to excessive data load.

Communicating consent and study information is challenging, especially for nonprofessionals and in case of a complex study design, such as TwiCs. The simplified text version of the study information as part of the eConsent application provides important assistance to the CHWs during recruitment. Limitations of our approach include the lack of multimedia communication support in the consent procedure. For future projects in similar settings, the additional use of other electronic communication aids such as videos or audio records should be considered, as this might further reduce the training needs for CHWs, ensure the completeness of information transmitted and increase the confidence of CHWs. Furthermore, the ComBaCaL app used for data collection and clinical decision support, does not include a feature for eConsent. Therefore, we implemented the eConsent via a separate application and then linked the two via file transfer from the eConsent application to the ComBaCaL app. This additional step requiring CHWs to switch between two applications limited user-friendliness and could be omitted by designing an application that contains all necessary features for data collection, clinical decision support and eConsent.

According to our knowledge based on a recently published scoping review,^10^ this is the first use of eConsent in a study using the TwiCs design. In our setting, where a complex consent is administered by more than hundred trained lay CHWs to over 16,000 participants in remote villages, eConsent is an essential tool for efficient study implementation and high-quality consent documentation.

## Conclusion

In summary, the ComBaCaL study has demonstrated that the use of eConsent may improve the efficiency and quality of consent documentation for large-scale pragmatic trials in remote settings. The use of eConsent has enabled lay CHWs to effectively communicate complex TwiCs consent information, has streamlined the trial implementation, and ensured the integrity and accessibility of consent records. The successful deployment of eConsent in our study underscores its value as a vital tool for facilitating high-quality research in remote settings. The integration of multimedia communication aids could further enhance the quality and ease of consent information communication.

## Supplemental Material

sj-docx-1-dhj-10.1177_20552076241288757 - Supplemental material for eConsent administered by Community Health Workers in a study using the Trials within Cohorts (TwiCs) design—Experiences from the Community-Based chronic Care Lesotho (ComBaCaL) project Supplemental material, sj-docx-1-dhj-10.1177_20552076241288757 for eConsent administered by Community Health Workers in a study using the Trials within Cohorts (TwiCs) design—Experiences from the Community-Based chronic Care Lesotho (ComBaCaL) project by Felix Gerber, Thesar Tahirsylaj, Thabo Ishmael Lejone, Tristan Lee, Giuliana Sanchez-Samaniego, Fabian Raeber, Sesale Masike, Ravi Gupta, Manthabiseng Molulela, Makhebe Khomolishoele, Mota Mota, Matumaole Bane, Mamoronts’ane Pauline Sematle, Retselisitsoe Makabateng, Jason Immanuel Browne, Jonas Wittwer, Dave Brian Basler, Kevin Kindler, Niklaus Daniel Labhardt and Alain Amstutz in DIGITAL HEALTH

## List of abbreviations

ComBaCaL: Community-Based chronic disease Care Lesotho
CHW: Community Health Worker
eConsent: electronic consent
PDF: portable document format
TwiCs: Trials within Cohorts
