# Supplementary material for: eConsent administered by Community Health Workers in a study using the Trials within Cohorts (TwiCs) design—Experiences from the Community-Based chronic Care Lesotho (ComBaCaL) project
Source: Digit Health. 2024 Oct 1;10:20552076241288757. doi: 10.1177/20552076241288757 (PMC11445768; doi:10.1177/20552076241288757)
Supplement: sj-docx-1-dhj-10.1177_20552076241288757 - Supplemental material for eConsent administered by Community Health Workers in a study using the Trials within Cohorts (TwiCs) design—Experiences from the Community-Based chronic Care Lesotho (ComBaCaL) project [file sj-docx-1-dhj-10.1177_20552076241288757.docx]

# Hello!

I am your Village Health Worker (VHW). I am part of a team conducting research on how to take better care of people with chronic diseases like high blood sugar and high blood pressure. I work closely with your nearby health facility.

We want to find out if delivering care in the village can be as good as or better than getting care in the health facility.

ComBaCaL stands for “Community-Based Chronic Care Lesotho” and is the name of the study I am going to explain to you and want to ask permission for.

If you don’t understand something, please stop me and I will take the time to explain it and answer all the questions you have. If you have questions later, you are free to contact me or the people listed below.

# Where is the ComBaCaL study taking place?

ComBaCaL will be in around 100 villages in Butha-Buthe and Mokhotlong.

# How does the study work?

- If you agree to join the study, I will use a tablet to collect information about you and your health.
- I will also conduct some tests for high blood pressure and high blood sugar (if you are at risk).
- During the next years, I will come back to visit you regularly.

# Asking Questions

I will ask you questions about:

- your current health, medical history and social life, including your schooling and what you do for work.
- questions that tell us if you are at risk for having diabetes or other medical problems, for example your diet, physical activity, smoking tobacco or taking alcohol.
- I may also ask questions about your mental well-being.
- I will also ask to look at your *bukana* to learn more about your health.

# Taking measurements about your health

- I will measure your height and weight.
- I will use an arm cuff and an automated machine to measure your blood pressure. Depending on what we learn about you at first, we may want to get a drop of blood from your finger, for example to check whether you could have high blood sugar.
- We may also ask to draw some blood from your arm for further examination; you can decide to not have this done. If we draw blood, we would keep it in Lesotho for up to five years for further analysis.

# What happens after the first visit and how long does the study last?

- If I find anything abnormal, such as high blood pressure or high blood sugar, I will offer to come back some days later to confirm the results and to discuss the options for treatment.
- If I do not find anything abnormal, I will visit you around twice a year.
- The project does not have a defined end date. If I leave the project, another VHW will start visiting you.

# How does the study work?

All villages will get the best available type of care but in different ways:

- We want to find out if we can deliver good quality care for high blood sugar and high blood pressure in different ways. For example, if we as VHWs can hand out medication so that you don’t have to go to the health facility too often.
- We study this by splitting the ComBaCaL villages into groups. We do this randomly (by chance), somewhat like rolling dice to make the decision. In this way, neither you nor I can influence which villages receive what.
- People in both groups will be followed and visited by us, the VHWs.
- While I will not be able to tell you which group you are in, I will explain any new intervention and service option to you. You can then always decide if you want to receive it or not.

# What is the benefit of being in the study?

- Finding a disease early can help prevent problems later.
- You will be helping us to find out how to take better care of chronic diseases in the communities of Lesotho and elsewhere.
- There is no payment or gifts for being in the study.

# Are there risks for being in the study?

- You might feel uncomfortable with some of the questions we ask.
- The finger prick can be uncomfortable.
- There can always be some risk when taking a new medication, but the risk is much smaller than the risk of not treating the disease.
- Otherwise, there are no risks involved.

# Do I have to be in the study?

It is your choice to be in the study and you can leave the study whenever you want. You are free to refuse any questions, measurements or interventions during the study.

I can still take care of you if you don’t want to be in the study, but it may be different care than if you were in the study. For example, if we find diabetes but you then do not want to be in the study, I will refer you to the health facility and will not be able follow your care in the village.

# Is my information private?

- I will record your private information on my tablet. But this info can only be accessed my me and a few members of the study team that need access to it for research purposes. They will keep your information confidential.
- We will not share information with health facility staff unless it is important for your health.
- Otherwise, we only share aggregated and anonymized data (that means people will not be able to identify you by name).

# What if I am not feeling well?

In case of medical problems, please come to me and tell me.

# Who is working in ComBaCaL?

ComBaCal is made up of people working for several different organizations:

- Ministry of Health in collaboration with your local health facility
- SolidarMed
- And partners from Europe (University of Basel, World Diabetes Fund, Swiss Development Corporation)

# If you have questions or concerns

You may always contact me directly. If you wish to speak to the people responsible for the study, you may contact the following persons at any time:

- Table with contact details of study team and representative of ethics committee (removed for publication)
